# Effects of Intravitreal Methotrexate Injection on Choroidal Structure in Intraocular Malignant Lymphoma and Identification of Prognostic Factors for Central Nervous System Lymphoma Development

**DOI:** 10.3390/biomedicines14010169

**Published:** 2026-01-13

**Authors:** Masayuki Yamada, Ryoji Yanai, Mariko Egawa, Yoshinori Mitamura

**Affiliations:** Department of Ophthalmology, Institute of Biomedical Sciences, Tokushima University Graduate School, Tokushima 770-8503, Japan; ma.yampi1430@gmail.com (M.Y.); egawa.m@tokushima-u.ac.jp (M.E.); ymita@tokushima-u.ac.jp (Y.M.)

**Keywords:** vitreoretinal lymphoma, intravitreal methotrexate, choroid, enhanced depth imaging OCT, central nervous system lymphoma

## Abstract

**Background**: Vitreoretinal lymphoma (VRL) often presents with features resembling uveitis and is commonly associated with central nervous system lymphoma (CNSL). Intravitreal methotrexate (IVMTX) is widely used as local therapy; however, objective markers for treatment response and prognosis remain limited. This study investigated choroidal structural changes after IVMTX via enhanced depth imaging optical coherence tomography (EDI-OCT) and explored prognostic indicators for subsequent CNSL development. **Methods**: This retrospective study included 18 patients (27 eyes) with VRL treated with IVMTX at Tokushima University Hospital between 2006 and 2021. EDI-OCT was conducted at baseline and at 1 and 3 months after IVMTX. Choroidal thickness and luminal and stromal areas were quantified through image binarization. The stromal/choroidal area (S/C) ratio and its association with CNSL onset were statistically analyzed. **Results**: The mean number of IVMTX injections administered over 3 months was 5.9 ± 1.3. Foveal retinal thickness did not significantly change, whereas foveal choroidal thickness significantly decreased from 275.8 ± 15.8 µm at baseline to 257.5 ± 14.7 µm at 1 month (*p* < 0.01). Total choroidal and stromal areas, particularly in the outer choroidal layer, were significantly decreased after IVMTX (*p* < 0.0001), whereas the luminal area in the inner layer modestly reduced (*p* < 0.05). The S/C ratio significantly declined at 1 month post-treatment (*p* < 0.001). Patients who developed CNSL within 2 years of VRL onset demonstrated higher baseline S/C ratios (*p* < 0.05). **Conclusions**: IVMTX induces measurable reductions in choroidal areas and stromal proportion, indicating decreased inflammatory infiltration. The baseline S/C ratio observed on EDI-OCT is a potential noninvasive biomarker of VRL activity and a prognostic indicator for early CNSL development.

## 1. Introduction

Vitreoretinal lymphoma (VRL) presents with ocular findings similar to uveitis, including anterior chamber cells, vitreous opacities, and retinal infiltration [1]. In VRL, lymphoma cells primarily infiltrate the vitreous, retina, or subretinal space, and form scattered, yellowish-white, patchy lesions that gradually coalesce and expand. Approximately 65–90% of VRL cases are associated with central nervous system lymphoma (CNSL), which significantly impacts prognosis [2]. Treatment relies on the presence of extraocular lesions, typically requiring a combination of systemic therapy (e.g., systemic chemotherapy, whole-brain radiation therapy, autologous transplantation) and local ocular treatment (e.g., intravitreal methotrexate [IVMTX] injections and ocular radiation therapy) [3]. However, appropriate indicators for assessing treatment efficacy in VRL remain unavailable, and thus identifying suitable treatment response markers and prognostic factors is an urgent priority.

Optical coherence tomography (OCT) enables detailed observation of the retina, whereas enhanced depth imaging OCT (EDI-OCT) visualizes the choroid. Sonoda et al. described a method for quantifying the choroidal lumen and stroma by converting EDI-OCT images into a two-tone scale [4]. In our previous study, we revealed that IVMTX treatment for VRL significantly reduces choroidal thickness but does not significantly change choroidal lumen area. The stromal area was significantly reduced; thus, we proposed that EDI-OCT could be used to assess disease activity in VRL [5].

This study examined the specific changes in choroidal structure after IVMTX treatment for VRL in a larger cohort. Choroidal S/C ratio on EDI-OCT was deemed suitable for assessing disease activity in VRL. Further, this study investigated the association between CNSL development and the choroidal S/C ratio in VRL.

To the best of our knowledge, no previous reports have described the association between changes in choroidal structure and CNSL. In this study, we report new findings on the association between choroidal structure and disease activity in VRL, including a more detailed analysis of choroidal structure by increasing the sample size and dividing the choroid into outer and inner layers. Further, we report on the potential of the choroidal S/C ratio as a biomarker for predicting CNSL occurrence.

## 2. Methods

This study included patients diagnosed with VRL who received IVMTX treatment and choroidal structure evaluations via EDI-OCT at baseline (before IVMTX) and at 1 and 3 months post-treatment (1M and 3M, respectively) at Tokushima University Hospital from May 2006 to March 2021. The Institutional Review Board of Tokushima University Hospital approved the research protocol, which adheres to the principles of the Declaration of Helsinki. Informed consent was waived due to the retrospective nature of the study.

Vitrectomy was done to facilitate vitreous biopsy and collection of vitreous samples before IVMTX for patients with suspected VRL. If vitrectomy was performed at another institution and vitreous samples could not be obtained at our hospital, aqueous humor samples were used instead, and ocular findings were considered for diagnosis. VRL was diagnosed based on the following: class IV or V cytology results of the vitreous sample, positive immunoglobulin H chain gene rearrangement, interleukin (IL)-10/IL-6 ratio of >1.0 in the vitreous fluid or aqueous humor, and assessment of ocular findings [6].

IVMTX (Pfizer, Kalamazoo, MI, USA) was injected into the vitreous cavity at 400 μg/0.1 mL per injection, administered weekly for the first month. The need for additional injections was identified based on the IL-10/IL-6 ratio in the aqueous humor and ocular findings. IVMTX administration was temporarily discontinued if IL-10/IL-6 levels became negative or the ocular findings improved and had low lesion activity, and the patient was observed. In such cases, recurrence was diagnosed based on ocular findings or IL-10/IL-6 levels in the aqueous humor, and this identified the need for additional IVMTX doses or to continue observation. Several treatment protocols for VRL have been reported, including the administration of IVMTX twice weekly initially [7,8]; however, considering patient burden and complications, including corneal epithelial damage, the aforementioned protocol in this study was used for treatment administration.

All patients underwent standard ophthalmic examinations, including visual acuity, intraocular pressure, slit-lamp microscopy (for the anterior segment, intermediate transparent media, and fundus), color fundus photography, autofluorescence imaging, and OCT imaging, before and after IVMTX administration, including re-administration. Fundus angiography and OCT angiography were performed as required.

**Figure 1:**
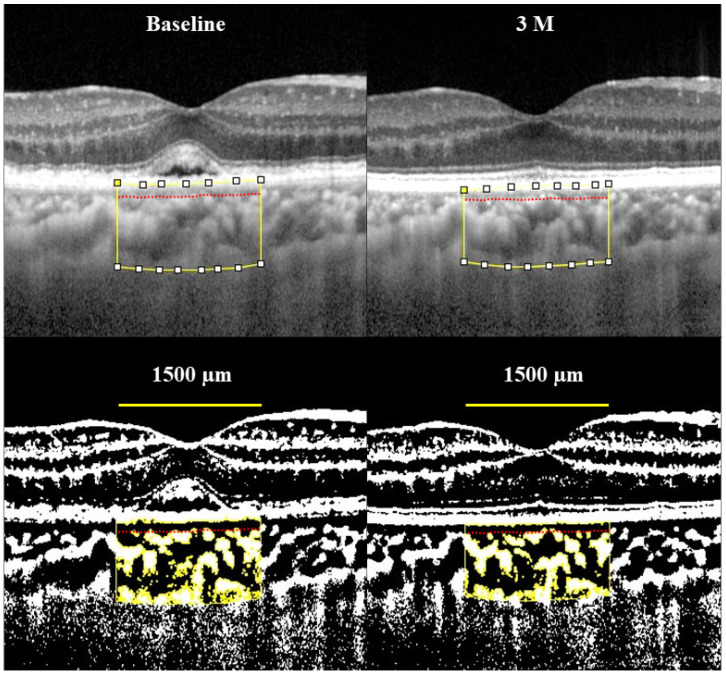
Representative case of binarized EDI-OCT images. The upper images show the original EDI-OCT scans, with their corresponding binarized images below. The luminal areas are displayed in black, and the stromal areas in white. From left to right are the images at baseline, after 1 month (1M), and after 3 months (3M) of treatment. The red line indicates the boundary between the inner and outer choroidal layers. A decrease in stromal area and choroidal thickness over time was seen after IVMTX treatment.

The Heidelberg Spectralis^®^ (Heidelberg Engineering, Heidelberg, Germany) was used to acquire EDI-OCT images. The examination area included the fovea centralis within a 1500-μm width. EDI-OCT images were binarized in the choroidal region. Choroidal thickness, choroidal area, vascular area, and stroma area were evaluated before and after IVMTX injection, separated into the choroid, choroidal intralayer, and choroidal extra-layer. The Niblack method was employed to perform binarization in the ImageJ image software (Version 0.71, NIH, Bethesda, MD, USA). Adopting the technique of Branchini et al., the choroidal inner layer was divided into the capillary and middle vascular layers, whereas the outer layer was split into the large vessel and episcleral layers [9]. The boundaries between the inner and outer layers, as well as between the outer layer and sclera, were identified manually. The choroidal region was selected using the ImageJ ROI Manager. The Ellipse Selection Tool from the ImageJ toolbar was used to randomly select three choroidal vessels, identifying their average reflectance. For reflectance calibration, choroidal vessels were randomly selected, and vessels of various sizes were included, not limited to large vessels alone. To minimize noise in the OCT images, the threshold tool was used to identify the choroidal lumen area, with bright pixels representing the stroma and dark areas denoting the lumen. The lumen and stroma areas were automatically calculated after summing the distance data for each pixel. Choroidal thickness and area were identified as the average of three measurements per eye (Figure 1). Cases were excluded if an accurate OCT assessment of choroidal structure was impossible or adequate image data was unavailable (i.e., severe vitreous opacity, large-scale retinal pigment epithelium detachment, high myopia). A single evaluator who was masked to the clinical outcomes performed all OCT dichotomization analyses.

Paired *t*-tests were used to investigate changes in the areas of the entire choroidal layer, lumen, and stroma, as well as changes in the choroidal stromal/choroidal area (S/C) ratio. Repeated analysis of variance (ANOVA) and corrected Bonferroni’s multiple comparison test were used to identify the significance of changes in these areas and the S/C ratio within the same eye, including recurrence, at three time points (before IVMTX, 1 month, and 3 months). We investigated changes in foveal retinal thickness (FRT), foveal choroidal thickness (FCT), and the areas of the entire choroidal layer and outer and inner choroidal layers, as well as changes in the areas of the lumen and stroma within each layer, in all 27 eyes with initial IVMTX without recurrence.

Furthermore, differences in choroidal structure were investigated between those who developed CNSL within 2 years versus after 2 years from the onset of VRL. The rationale for distinguishing “early” from “late” CNSL development using a 2-year cutoff was based on previous literature indicating that CNSL most frequently develops within the first 2 years after VRL diagnosis [10]. One of the cases with an extraocular onset was excluded due to a history of recent chemotherapy, which may have influenced CNSL development. CNSL development is a patient-level outcome, whereas choroidal structural analyses were conducted at the eye level. VRL frequently demonstrates bilateral or symmetric ocular involvement, and baseline choroidal structural parameters were generally comparable between eyes within the same patient in this cohort. Therefore, each eye was considered an analytical unit to assess local ocular biomarkers that reflect disease activity.

Previous studies have reported that the total choroidal, stromal, and luminal areas significantly decrease with increasing age and longer axial length [11]. However, axial length measurements were not available for all cases in this study. Therefore, analysis of covariance (ANCOVA) was conducted using age and refraction value (as a substitute for axial length) as covariates to adjust for the effects of aging and axial length. To address potential bias related to within-patient correlation, ANCOVA was used to adjust comparisons associated with CNSL development, with age and refraction as covariates.

Cases of high myopia with extremely long axial lengths were excluded from the analysis, although adjustment for axial length was not performed. *p*-values of <0.05 were considered statistically significant.

## 3. Results

The study included 18 patients (27 eyes; comprising 10 males and 8 females) with a mean age of 68.6 ± 12.9 years (range, 46–92) (Table 1). Ocular onset was present in 15 cases. Extraocular onset was observed in three cases, among which the initial presentation was diffuse large B-cell lymphoma (DLBCL) of the skin (*n* = 1) and intravascular DLBCL (*n* = 2). Among all 18 patients, the initial presentation was unilateral in 9 cases and bilateral in 9 cases, with 4 cases progressing from unilateral to bilateral disease.

Cytokine measurements in the anterior or posterior chamber fluid revealed an IL-10/IL-6 ratio of >1 in 24 eyes (88.9%) and <1 in 3 eyes (11.1%). Out of 27 eyes, 16 (59.3%) had retinal/subretinal lesions, whereas 11 (40.7%) had anterior chamber cells or vitreous opacities without retinal/subretinal lesions. Recurrence was defined as worsening ocular findings (e.g., anterior chamber cells, vitreous opacities, retinal/subretinal lesions) at least 3 months after the final IVMTX dose. Recurrence was observed in 12 eyes (44.4%), with a maximum of three recurrences per eye, whereas 15 eyes (55.6%) demonstrated no recurrence. Regarding CNSL, 18 eyes (66.7%) had a history of CNSL or developed CNSL within 2 years of VRL onset, whereas 9 eyes (33.3%) had no CNSL or developed it after 2 years.

**Figure 2:**
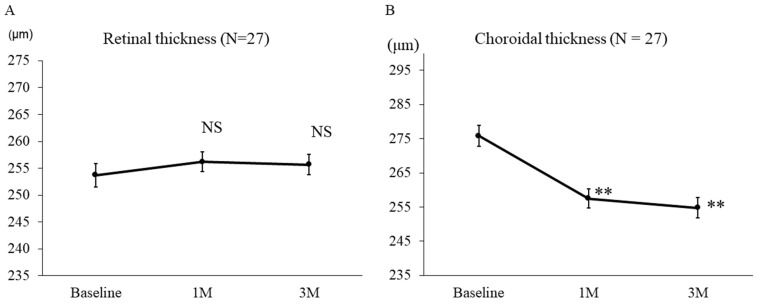
Effect of IVMTX on retinal and choroidal thickness in VRL. After IVMTX, foveal retinal thickness remained unchanged, but foveal choroidal thickness exhibited a significantly more pronounced decrease after 1 and 3 months. **: *p* < 0.01 (Repeated ANOVA & Bonferroni’s multiple comparison test), NS: not significant.

The mean number of IVMTX injections throughout the study period was 5.93 ± 1.30 (range, 2–7). Figure 1 illustrates changes in FRT and FCT after IVMTX. No significant change in FRT was observed from baseline (253.6 ± 11.6 μm) to after IVMTX (1M: 256.2 ± 9.8 μm, *p* = 0.49, 3M: 255.7 ± 9.9 μm, *p* = 0.62) (Figure 2A). In contrast, FCT decreased significantly compared with baseline (275.8 ± 15.8 μm) starting at 1 month after IVMTX (1M: 257.5 ± 14.7 μm, *p* = 0.0027; 3M: 254.8 ± 15.4 μm, *p* = 0.0016) (Figure 2B).

**Figure 3:**
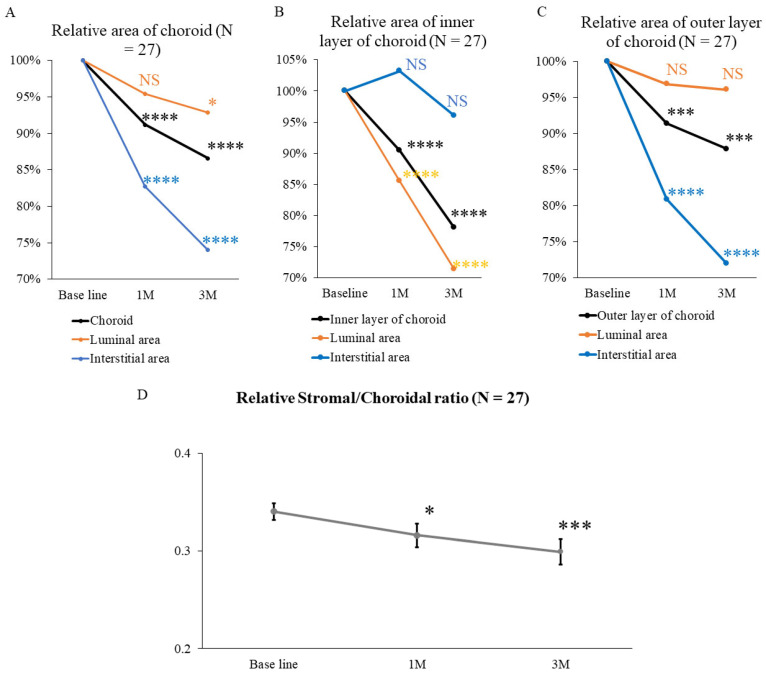
Effects of IVMTX on the total choroidal area, luminal area, and stromal area in VRL. (**A**) Relative area of choroid. IVMTX significantly reduced the relative choroidal area and the relative stromal area at 1 and 3 months after treatment, whereas the relative luminal area significantly decreased at 3 months after treatment. (**B**) Relative area of the inner layer of the choroid. IVMTX significantly reduced both the relative total choroidal inner layer and luminal areas, but the relative stromal area remained unchanged. (**C**) Relative area of the outer layer of the choroid. IVMTX significantly reduced both the total choroidal outer layer and stromal areas, but the relative luminal area remained unchanged. *: *p* < 0.05, ***: *p <* 0.001, ****: *p <* 0.0001 (Repeated ANOVA & corrected Bonferroni’s multiple comparison test), NS: not significant. (**D**) Relative stromal/choroidal ratio. IVMTX significantly reduced the choroidal stromal/choroidal area ratio at 1 and 3 months after treatment. *: *p* < 0.05, ***: *p <* 0.001 (Repeated ANOVA & Bonferroni’s multiple comparison test).

To analyze changes in choroidal structure induced by IVMTX in detail, changes in relative luminal and stromal areas were compared with baseline values. Choroidal and stromal areas significantly decreased 1 month after IVMTX (*p* < 0.0001). Luminal area remained similar 1 month after IVMTX (*p* = 0.07), but decreased significantly at 3 months after IVMTX (*p* < 0.05) (Figure 3A). Within the inner choroidal layer, the total inner layer and luminal areas were both significantly reduced after IVMTX (*p* < 0.0001), whereas the stromal area remained (*p* = 0.88) (Figure 3B). Conversely, in the outer choroidal layer, both the total outer layer and stromal areas were significantly reduced after IVMTX (*p* < 0.001), whereas the luminal area remained (*p* = 0.21) (Figure 3C).

The outer choroidal layer contains macrophages, dendritic cells, and other immune cells, and serves as a pathway for immune cells. T S/C ratio was calculated to identify whether this area could serve as an indicator of VRL disease activity (Figure 3D). The S/C ratio significantly decreased after 1 month of starting IVMTX (1M: 0.32. ± 0.01, *p* < 0.05; 3M: 0.30 ± 0.01, *p* < 0.001).

**Figure 4:**
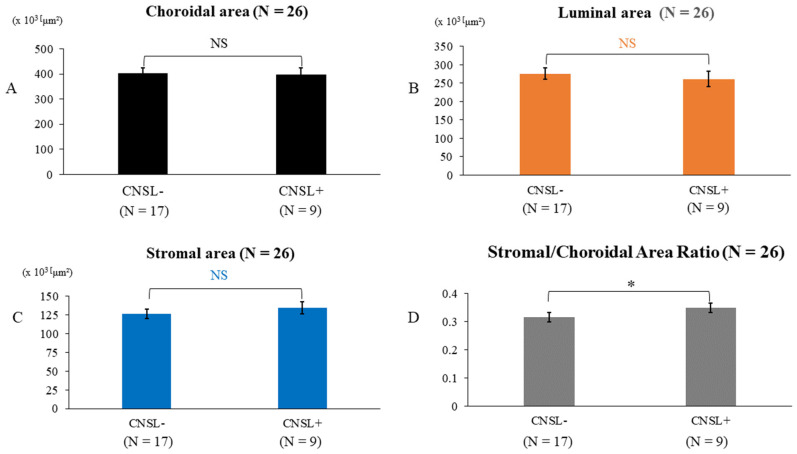
Total choroidal area, luminal area, stromal area, and stromal/choroidal area ratio after the development of CNSL. The development of CNSL did not alter total choroidal area (**A**), vascular area (**B**), or stromal area (**C**), but the stromal/choroidal ratio was significantly increased (**D**). *: *p* < 0.05 (ANCOVA), NS: not significant.

To examine choroidal structures associated with the onset of CNSL, the baseline total choroidal area, vascular area, stromal area, and S/C ratio were compared between those who developed CNSL within 2 years and those who did not. No changes in total choroidal area (*p* = 0.83) (Figure 4A), luminal area (*p* = 0.86) (Figure 4B), or stromal area (*p* = 0.37) (Figure 4C) were observed between the two groups. However, the S/C ratio was significantly higher among patients who developed CNSL within 2 years of VRL diagnosis (adjusted mean difference, 3.13%; 95% confidence interval, 0.37–5.90; F (1,22) = 5.51; *p* = 0.028; partial η^2^ = 0.20) (Figure 4D). Therefore, the S/C ratio at VRL diagnosis may serve as a prognostic indicator, specifically for CNSL development within 2 years.

## 4. Discussion

This study investigated changes in choroidal structure on EDI-OCT before and after IVMTX treatment for VRL. After IVMTX initiation, the FRT remained unchanged, whereas the FCT significantly decreased after 1 month. Choroidal analysis revealed substantial reductions in the luminal area of the inner layer and stromal area of the outer layer, alongside a decrease in S/C ratio. Furthermore, cases with a higher S/C ratio at the onset of VRL tended to develop CNSL within 2 years, indicating that this ratio may serve as a prognostic indicator.

The choroid thickens during the active phase and becomes relatively thinner during the inactive phase in uveitis with retinal lesions as the primary manifestation, including Behçet’s disease or acute zonal occult outer retinopathy [12,13]. In VRL, choroidal thickness and area similarly decrease post-treatment [14], likely because reactive inflammatory cells infiltrate the choroidal stroma in VRL, thereby increasing the stromal area, which decreases with treatment [15]. Moreover, lymphoma cells infiltrate the vitreous and the space between the retinal pigment epithelium and Bruch’s membrane. A previous study revealed that lymphoma cells rarely infiltrated beyond the retinal pigment epithelium into the choroid when injected into the vitreous of mice [16]. In a choroidal biopsy from a woman who was blinded due to VRL, lymphoma cells and necrotic tissue were observed in the subretinal space but not the choroid, whereas inflammatory cells infiltrated the choroid [17]. Consequently, choroidal area reduction and S/C ratio after IVMTX in this study are attributed not to decreased lymphoma cells but rather to reduced inflammatory cells that infiltrate the choroidal stroma due to the resolution of inflammation. Furthermore, the retina itself had minimal infiltration of lymphoma cells or inflammatory cells; thus, retinal thickness at the fovea likely remained unchanged during treatment.

The association between choroidal structure and CNSL/VRL has not been previously investigated. Moreover, the mechanisms by which primary VRL develops into CNSL, or how CNSL itself generates VRL, remain unclear. Interestingly, a previous study revealed that patients with VRL without CNSL may have increased IL-10 in cerebrospinal fluid and potential infiltration despite having no visible brain lesions on imaging [18]. This raises the possibility of micro-infiltration of lymphoma cells into the retina or optic nerve, but the exact mechanism behind this remains unclear. In this study, patients with an interval of less than 2 years between the onset of VRL and CNSL demonstrated a significantly higher proportion of choroidal stroma. However, no change was observed in the area of the choroid itself, including the stromal and lumen area, versus those with an interval of 2 years or more, or those who did not develop CNSL. Higher VRL activity and greater inflammatory cell infiltration into the choroidal stromal area may have increased the S/C ratio in patients with VRL who developed CNSL shortly thereafter. Thus, the S/C ratio in patients with VRL can help predict the onset of CNSL and prognosticate patients, making it a more sensitive indicator than evaluating the choroidal area itself.

IVMTX significantly reduced the stromal area of the entire choroid and the outer choroidal layer. Treatment reduces the inflammatory cells infiltrating the outer choroidal stroma; thus, this may decrease the outer choroidal stromal area, thereby reducing the overall choroidal stroma area. In the inner choroidal layer, IVMTX significantly reduced the overall choroidal and luminal areas. The choroidal vessels dilate during the acute phase of acute anterior uveitis, including HLA-B27-associated anterior uveitis, and improve post-treatment [13,19,20]. IVMTX improved the vasodilation of the choroidal inner layer; thus, the luminal area of the inner layer decreased, thereby potentially reducing the overall choroidal luminal area. Thus, in VRL, IVMTX treatment can reduce choroidal area through two mechanisms: (1) improving vasodilation by reducing inflammation, and (2) decreasing inflammatory cells that infiltrate the stroma. Understanding these mechanisms, alongside S/C ratio assessment, can help in evaluating treatment efficacy.

This study has several limitations. First, choroidal structural analyses were conducted at the eye level, whereas CNSL development represents a patient-level outcome. Bilateral or symmetric ocular involvement was common in our cohort; however, this approach may have introduced intra-subject correlation. Future studies with larger sample sizes are recommended to consider patient-level analyses or mixed-effects models to further validate the association between choroidal biomarkers and CNSL development. Second, the sample size is small. Further studies with a larger sample size and investigations for additional parameters that show significant changes could improve the diagnostic value of EDI-OCT during VRL treatment. Third, axial length was not measured in all cases in this study, despite its important correlation with choroidal area [11]. To mitigate this limitation, correction was limited to age and refraction value as a substitute for axial length, and high myopia cases with presumed excessive axial length were excluded. Notably, previous reports have identified gender-based differences in choroidal area [11,21], indicating that correction for gender and accurately measured axial length may have been necessary to produce more accurate data. Finally, regarding CNSL onset, the correlation with systemic inflammatory markers, such as IL-10 in the cerebrospinal fluid, needs to be evaluated to accurately capture CNSL onset.

VRL had a high recurrence rate of 44% in this study. The further evaluation of the choroidal area and the S/C ratio could help predict recurrence and disease onset in the unaffected eye.

Recent reports have used noninvasive devices, including OCT and OCTA, to evaluate the choroid and assess the activity of various uveitis-related conditions [22,23]. This practice provides a valuable means to understand the current status of patients with VRL and identify future treatment plans without a significant burden on the patient.

## 5. Conclusions

Among patients with VRL, noninvasive imaging examination with EDI-OCT revealed that choroidal structures change with IVMTX treatment. These changes may serve as indicators for the onset or recurrence of VRL. Furthermore, the S/C area ratio at the onset of VRL may serve as a potential prognostic biomarker and indicator for CNSL development.

## Figures and Tables

**Table 1:** Demographic information and characteristics.

Participants		18
Age		68.6 ± 12.9
Pathological	GCB	5 (27.8%)
Sex	Male	10 (55.6%)
	Female	8 (44.4%)
Primary lesion	Eyes	15 (83.3%)
	Extra-Eyes	3 (16.7%)
Distribution	Bilateral	9 (50%)
	Unilateral	9 (51%)
Eye number		27
IL-10/IL-6	IL-10/IL-6 > 1	24 (88.9%)
	IL-10/IL-6 < 1	3 (11.1%)
Recurrence	+	12 (44.4%)
	−	15 (55.6%)
CNLS onset within 2 years	+	18 (66.7%)
	−	9 (33.3%)

CNSL: central nervous system lymphoma; GCB: Germinal Center B-cell.

## Data Availability

The original contributions presented in this study are included in the article. Further inquiries can be directed to the corresponding author.

## References

[1] Gill M.K., Jampol L.M. (2001). Variations in the presentation of primary intraocular lymphoma: Case reports and a review. Surv. Ophthalmol..

[2] Chan C.C., Sen H.N. (2013). Current concepts in diagnosing and managing primary vitreoretinal (intraocular) lymphoma. Discov. Med..

[3] Taoka K., Yamamoto G., Kaburaki T., Takahashi T., Araie M., Kurokawa M. (2012). Treatment of primary intraocular lymphoma with rituximab, high-dose methotrexate, procarbazine, and vincristine chemotherapy, reduced whole-brain radiotherapy, and local ocular therapy. Br. J. Haematol..

[4] Sonoda S., Sakamoto T., Yamashita T., Shirasawa M., Uchino E., Terasaki H., Tomita M. (2014). Choroidal structure in normal eyes and after photodynamic therapy determined by binarization of optical coherence tomographic images. Investig. Opthalmol. Vis. Sci..

[5] Egawa M., Mitamura Y., Sano H., Akaiwa K., Niki M., Semba K., Sonoda S., Sakamoto T. (2015). Changes of choroidal structure after treatment for primary intraocular lymphoma: Retrospective, observational case series. BMC Ophthalmol..

[6] Takase H., Arai A., Iwasaki Y., Akaiwa K., Niki M., Semba K., Sonoda S., Sakamoto T. (2022). Challenges in the diagnosis and management of vitreoretinal lymphoma—Clinical and basic approaches. Prog. Retin. Eye Res..

[7] Frenkel S., Hendler K., Siegal T., Shalom E., Pe’er J. (2008). Intravitreal methotrexate for treating vitreoretinal lymphoma: 10 years of experience. Br. J. Opthalmol..

[8] Hsu C.-Y., Hou H.-A., Lin C.-P., Lee Y.-J., Hsu W.-F., Yeh P.-T. (2022). Clinical outcomes of intravitreal methotrexate injection protocol with a reduced initial frequency for intraocular lymphoma. J. Formos. Med. Assoc..

[9] Branchini L.A., Adhi M., Regatieri C.V., Nandakumar N., Liu J.J., Laver N., Fujimoto J.G., Duker J.S. (2013). Analysis of choroidal morphologic features and vasculature in healthy eyes using spectral-domain optical coherence tomography. Ophthalmology.

[10] Grimm S.A., Pulido J.S., Jahnke K., Schiff D., Hall A.J., Shenkier T.N., Siegal T., Doolittle N.D., Batchelor T., Herrlinger U. (2007). Primary intraocular lymphoma: An international primary central nervous system lymphoma collaborative group report. Ann. Oncol..

[11] Sonoda S., Sakamoto T., Yamashita T., Uchino E., Kawano H., Yoshihara N., Terasaki H., Shirasawa M., Tomita M., Ishibashi T. (2015). Luminal and stromal areas of choroid determined by binarization method of optical coherence tomographic images. Am. J. Ophthalmol..

[12] Ishikawa S., Taguchi M., Muraoka T., Sakurai Y., Kanda T., Takeuchi M. (2014). Changes in subfoveal choroidal thickness associated with uveitis activity in patients with Behçet’s disease. Br. J. Ophthalmol..

[13] Maehara H., Sekiryu T., Sugano Y., Maruko I. (2019). Choroidal thickness changes in acute zonal occult outer retinopathy. Retina.

[14] Kim R.-Y., Park J.H., Kim M., Park Y.-G., Cho S.-G. (2021). Changes in choroidal vascular structure from vitreoretinal lymphoma and the intraocular cytokine level associated with clinical resolution after intravitreal methotrexate treatment. PLoS ONE.

[15] Lopez J.S., Chan C.-C., Burnier M., Rubin B., Nussenblatt R.B. (1991). Immunohistochemistry findings in primary intraocular lymphoma. Am. J. Ophthalmol..

[16] Chan C.C., Fischette M., Shen D., Mahesh S.P., Nussenblatt R.B., Hochman J. (2005). Murine model of primary intraocular lymphoma. Investig. Opthalmol. Vis. Sci..

[17] Read R.W., Zamir E., Rao N. (2002). Neoplastic masquerade syndromes. Surv. Opthalmol..

[18] Nguyen-Them L., Costopoulos M., Tanguy M.L., Houillier C., Choquet S., Benanni H., Elias-Shamieh R., Armand M., Faivre G., Glaisner S. (2016). The CSF IL-10 concentration is an effective diagnostic marker in immunocompetent primary CNS lymphoma and a potential prognostic biomarker in treatment-responsive patients. Eur. J. Cancer.

[19] Kim M., Kim R.-Y., Park Y.-H. (2019). Choroidal vascularity index and choroidal thickness in human leukocyte antigen-B27-associated uveitis. Ocul. Immunol. Inflamm..

[20] Balci S., Turan-Vural E. (2020). Evaluation of changes in choroidal vascularity during acute anterior uveitis attack in patients with ankylosing spondylitis by using binarization of EDI-optical coherence tomography images. Photodiagnosis Photodyn. Ther..

[21] Li X.Q., Larsen M., Munch I.C. (2011). Subfoveal choroidal thickness in relation to sex and axial length in 93 Danish university students. Investig. Ophthalmol. Vis. Sci..

[22] Dhirachaikulpanich D., Chanthongdee K., Zheng Y., Beare N.A.V. (2023). A systematic review of OCT and OCT angiography in retinal vasculitis. J. Ophthalmic Inflamm. Infect..

[23] Herbort C.P., Takeuchi M., Papasavvas I., Tugal-Tutkun I., Hedayatfar A., Usui Y., Ozdal P.C., Urzua C.A. (2023). Optical coherence tomography angiography (OCT-A) in uveitis: A literature review and a reassessment of its real role. Diagnostics.

